# Antecedents of Residents’ Pro-tourism Behavioral Intention: Place Image, Place Attachment, and Attitude

**DOI:** 10.3389/fpsyg.2019.02349

**Published:** 2019-10-23

**Authors:** Ke Shen, Chuan Geng, Xinwei Su

**Affiliations:** ^1^Faculty of International Tourism and Management, City University of Macau, Macau, China; ^2^School of Tourism, Huangshan University, Huangshan, China; ^3^Faculty of Business, City University of Macau, Macau, China; ^4^School of Tourism, Liming Vocational University, Quanzhou, China

**Keywords:** place image, place attachment, attitude, residents, pro-tourism behavioral intention

## Abstract

Few prior studies have investigated place image from the residents’ perspective, how this and residents’ place attachment influence attitude to tourism, and consequent reactions. Accordingly, this study aims to develop a model for local residents’ pro-tourism behavioral intention and to discover the relationships between constructs. Analysis was based on a sample of 370 residents in Huangshan City, China. Results indicate that residents’ attitude to tourism positively affects their pro-tourism behavioral intention. Residents’ place image is found to positively relate to place attachment and attitude to tourism, while place attachment is also related to attitude and pro-tourism behavioral intention. In addition, attitude to tourism mediates place image’s and place attachment’s respective relationships with pro-tourism behavioral intention. Lastly, place image indirectly impacts residents’ attitude to tourism and pro-tourism behavioral intention through place attachment. However, the positive relationship between place image and pro-tourism behavioral intention is not supported. Theoretical and practical implications are discussed.

## Introduction

With the development of urbanization and improvement in living standards, traveling has become an important leisure pursuit for relaxation, especially for people living in urban areas. In choosing tourism destinations, people usually prioritize small cities, towns, and village with mountains, lakes, and other wild places, which “represent escape locations that offer excitement, stimulation, and potential adventure” (Beedie and Hudson, 2003, p. 625). Tourists inevitably cause interaction with local residents, who are directly or indirectly impacted positively and/or negatively (Yoon et al., 2001). As one of the stakeholders, local residents have been becoming increasingly important in the destination, and their support is regarded as a significant precondition for a destination’s tourism sustainability (Agapito et al., 2010; Sinclair-Maragh and Gursoy, 2016; Ribeiro et al., 2017). Nunkoo et al. (2013, p. 6) even argue that residents’ “participation and cooperation was crucial factor for sustainable tourism development.” Although some empirical studies have explored residents’ attitude and reaction to local tourism development, few consider residents’ place image and place attachment as antecedents (Stylidis, 2015; Eusébio et al., 2018).

Place image has been proven to play an important role in the sustainability of places as tourist destinations (Ritchie and Crouch, 2000). However, most prior studies have only considered place image from the perspective of tourists or consumers (Silva et al., 2013; Chew and Jahari, 2014; Özdemir and Şimşek, 2015; Tsai, 2015; Kock et al., 2016; Martín-Santana et al., 2017; Song et al., 2017; Stylos et al., 2017), with only a handful concentrating on residents’ perspective (Stylidis et al., 2016, 2018). Moreover, most of the latter studies only compare the different attributes forming place image and perception of place image from perspectives of tourists and residents (Moreno Gil and Ritchie, 2008; Agapito et al., 2010; Papadimitriou et al., 2015; Stylidis et al., 2015, 2017; Ku and Mak, 2017; Slak and Williams, 2018). Thus, research on residents’ place image is seriously limited.

Although residents’ place attachment is among “the most prominent non-economic constructs used to explain why residents support or oppose tourism development” (Strzelecka et al., 2017, p. 61), the link between residents’ place attachment and their attitude to tourism development remains little understood (Eusébio et al., 2018). In addition, few researchers have explored the relationship between place image and place attachment (Stylidis, 2018) and even fewer have investigated the influence of place image and place attachment on other variables, such as attitude and reaction to tourism development (Stylidis et al., 2014). To the best of our knowledge, only five prior studies have used structure equation model (SEM) to demonstrate the effect of residents’ place image and place attachment on other variables (Schroeder, 1996; Ramkissoon and Nunkoo, 2011; Stylidis et al., 2014, 2018; Stylidis, 2015).

To fill this gap, our study aims to construct a theoretical model and explore the influence of residents’ place image and place attachment on their attitude to tourism and pro-tourism behavioral intention. Our findings should yield beneficial insight into the place as tourist destination, especially regarding sustainability.

## Literature Review and Hypothesis Formulation

### Residents’ Pro-tourism Behavioral Intention

As one of the stakeholders in tourism destinations, local residents’ support is increasingly important for their sustainability. Some scholars regard residents’ “support” in terms of attitude (Gursoy et al., 2002), while others consider it as behavior intention (Jackson and Inbakaran, 2006; Kwon and Vogt, 2010). Accordingly, this study considers residents’ attitude toward the impacts of tourism development and their pro-tourism behavioral intention. Given the importance of residents’ support for tourism development, many studies have investigated why residents support or oppose. Most of these studies are atheoretical, choosing not to utilize the extant framework to explain residents’ support. In the studies that are theoretical, social exchange theory (SET) is most frequently used to explain residents’ support for tourism development, followed by the Tourist Area Life Cycle (TALC), the theory of reasoned action (TRA), and the Irridex model (Nunkoo et al., 2013). In terms of antecedents of residents’ pro-tourism behavioral intention, the following are often examined: attitude, personal benefit, state of local economy, welcome tourists behavior, identity, subjective norm, gender, and tourism-related business (Jackson and Inbakaran, 2006; Chen and Raab, 2012; Nunkoo and Gursoy, 2012; Wang and Xu, 2015; Homsud, 2017; Martín et al., 2017; Ribeiro et al., 2017).

In a nutshell, residents’ pro-tourism behavioral intention plays an important role in determining the sustainability or even success of a tourist destination. Therefore, related study findings will be increasingly significant in the future.

### Residents’ Attitude to Tourism

Attitude, a very important concept rooted in social psychology, refers to a predisposition to place, people, behaviors, and other aspects of individual environment (Gu and Ryan, 2008). Based on this definition, attitude and behavior (intention) are believed to be closely related. However, the influence of residents’ attitude to tourism on their subsequent behavior is still under-examined (Sharpley, 2014).

Tourism is usually regarded as a double-edged sword for the destination. On the one hand, tourism can generate benefits including job opportunity, revenue, life satisfaction, investment in infrastructure, and preserving local culture. On the other hand, tourism can generate negative impacts, including increases of living costs, pollution, and even crime. Consequently, residents may hold a positive or negative attitude to tourism. However, few studies have explored the antecedents of residents’ attitude to tourism (Eusébio et al., 2018), and most of this research has been conducted in developed countries (Sharpley, 2014). Therefore, it is important to study on residents’ attitude in developing countries, such as China.

According to Lepp (2007) and Nunkoo and Gursoy (2012), residents’ pro-tourism behavioral intention is mostly affected by residents’ attitude to tourism. Martín et al. (2017) report that residents’ attitude could be categorized into two parts: attitude toward tourism and attitude toward tourists; they found that both positively influenced behavioral support for tourism development. Moghavvemi et al. (2017), taking two Malaysian tourist destinations as study sites, found a positive relationship between residents’ attitude and their support for tourism development. Based on SET and TRA, Chen and Raab (2012) constructed an integrated model and confirmed the positive relationship between residents’ attitude and their support for the community tourism. With a case study of Hua-Hin Prachubkirikhan, Homsud (2017) found that residents from both developed and developing counties preferred to hold pro-tourism behavior. De Leeuw et al. (2015) found that attitude to tourism was positively related to pro-environmental behavior among high school students. More evidences could be found in the attitude-behavior theories from social and environmental psychology, such as TRA, the theory of planned behavior (TPB), the norm activation model, the trans-theoretical model, and SET.

Based on the above discussion, we hypothesize:

H1: Residents’ attitude to tourism is positively related to their pro-tourism behavioral intention.

### Residents’ Place Attachment

Place attachment, a kind of bonding or connection with a particular place, is rooted in environmental psychology (Altman and Low, 1992). Researchers in this field have conducted abundant studies to conceptualize, understand, and measure individual-place bonding (Chen et al., 2014).

Previous studies have concentrated on the conceptualization and determinants of place attachment (Chen, 2018). Some researchers regard it as a multi-dimension variable (Chen, 2018; Huang et al., 2018), while Vong (2015); Scannell and Gifford (2016), and Tournois and Djeric (2018) consider place attachment a single-dimension variable. In recent research, place attachment as a two-dimension variable comprising place identity and place dependence is widely accepted in the context of environmental psychology and tourism management (Chen et al., 2014). Researchers have attempted to psychometrically distinguish between these two dimensions as functional goals (place dependence) and symbolic meanings (place identity) (Raymond et al., 2017). Because our study’s participants include not only native inhabitants but also some immigrants undertaking short-term work in the study area, place identity seems unsuitable. Therefore, we adopt place attachment as a single-dimension variable.

The main determinants of place attachment are experience, involvement, and satisfaction (Buonincontri et al., 2017; Ramkissoon and Mavondo, 2017; Chen, 2018). Beyond the emotional connection between human beings and landscapes, “they also have a deep and complex attachment that is expressed through emotional and behavioral actions” (Bricker and Kerstetter, 2000, p. 234). Once place attachment is established, it will influence individual perception and consequent behaviors toward the place, such as satisfaction, loyalty, attitude, and pro-environmental behavior (Su and Qian, 2012). These demonstrate the relationships between place attachment and both attitude to tourism and behavior. However, place attachment has received limited attention as a determinant of residents’ attitude to tourism (Eusébio et al., 2018), and there is no consensus on the nature of this relationship (Tournois and Djeric, 2018). Some studies have found that place attachment is positively related to both residents’ attitude to tourism and their pro-tourism behavioral intention; however, others have found that place attachment negatively affects both variables.

In Eusébio et al.’s (2018) study of Boa Vista Island, Cape Verde, residents’ place attachment positively influences not only the perceived impact of but also attitude to tourism. Gu and Ryan (2008) also found that residents’ place attachment positively influenced their attitude in a study of Shi Cha Hai hutong, Beijing. Based on SET, Choi and Murray (2010, p. 588) found that “highly attached residents appeared to evaluate additional tourism development positively.” In their investigation of 358 Sydney residents, Chen and Dwyer (2017) found that place attachment was positively related to behavior, including WOM, ambassador behavior, and participation. Through research in Jiuzhaigou, China, Zhang et al. (2017) found that place attachment was the strongest influencing factor of local residents’ environmental conservation behavior, surpassing awareness of environmental consequences and values. In Ning et al.’s (2014) study of 691 residents in Sydney and Shanghai, place attachment positively influenced their WOM behaviors.

Though abundant research has revealed the impact of place attachment on attitude to tourism and behavioral intention, few studies have investigated the indirect impact of residents’ place attachment on behavioral intention through attitude. Our study tests this relationship using PLS-SEM, which is suitable for exploratory research and theory development (Hair et al., 2012; Ringle et al., 2012). So, it is reasonable to assume the indirect influence of place attachment on behavioral intention through attitude.

Based on the above discussion, we hypothesize:

H2: Residents’ place attachment is positively related to their attitude to tourism.
H3: Residents’ place attachment is positively related to their pro-tourism behavioral intention.
H3a: Residents’ attitude to tourism positively mediates the relationship between place attachment and pro-tourism behavioral intention.

### Residents’ Place Image

In the context of tourism, place image is defined as a set of impressions, ideas, expectations, and emotional thoughts toward a place as a tourist destination (Assaker, 2014) and is characterized as a psychographic segmentation variable (Stylidis et al., 2018). Although some scholars prefer to use “destination image,” this is essentially synonymous with place image in the context of tourism if the place is a tourist destination.

After four decades of research, the antecedents, consequences, components, formation, and categories of place image are well developed. The main antecedents of place image include the time spent searching for information (Baloglu and McCleary, 1999a, b), involvement and visit intensity (Martín-Santana et al., 2017), destination imagery and affect (Kock et al., 2016), satisfaction, perceived price and quality (Özdemir and Şimşek, 2015), perceived tourism development (Silva et al., 2013), and motivation and information source (Moreno Gil and Ritchie, 2008). Regarding the consequence, place image can influence the perceived impacts of tourism (positive or negative), revisit intention, recommendation, satisfaction, loyalty, WOM behavioral intention, perceived quality, attitude, and support behavioral intention (Papadimitriou et al., 2015; Tsai, 2015; Kock et al., 2016; Martín-Santana et al., 2017; Song et al., 2017; Stylidis, 2017; Stylos et al., 2017; Stylidis et al., 2018). How place image influences people’s attitude to tourism and behavior has been investigated in environmental psychology (Lynch, 1960), geography (Bolton, 1992), and marketing studies (Elliot et al., 2011). Environmental psychology research has long acknowledged the significant impact of place image on residents’ attitude to tourism and behaviors (Stylidis, 2015), but its influence in the context of tourism is till scarce (Stylidis, 2017).

Ramkissoon and Nunkoo (2011) found that the place image of Mauritian residents positively influenced their support for tourism. Schroeder’s (1996) study of North Dakotan residents found that those holding a positive place image were more supportive of tourism and preferred to travel within the state. With a sample of 466 urban residents in Belgrade, Tournois and Djeric (2018) found that place image positively and directly impacted support for tourism development. In Stylidis et al.’s (2014) study in Kavala (Greece), residents’ place image positively affected their support for tourism development. In a later study of Eilat (Israel), Stylidis (2018) also found that residents with the most favorable place image (*Appreciators*) exhibited higher support for tourism than those with the least favorable image (*the Critical*). In another study of Eilat, Stylidis et al. (2017) found that residents’ overall place image exerted a positive and direct influence on their intention to recommend. Tourists’ place image has also been found to positively affect their intention to revisit a tourist destination (Stylos et al., 2017). Similarly, destination image affected Chinese college students’ attitude and intention to travel to Japan (Park et al., 2017).

Further evidence could be found with respect to the conative image component. As one of the three parts of place image, conative image “is analogous to behavior because it is the action component” (Gartner, 1994, p. 196). Some scholars even regard them as synonymous (Chen and Sambath, 2013; King et al., 2015; Stylidis et al., 2015). It can, thus, be inferred that place image and behavioral intention are likely to be closely related.

To the best of our knowledge, no research has tested the indirect impact of residents’ place image on behavioral intention via attitude to tourism. Our study tests this relationship using PLS-SEM. It is reasonable to assume the indirect influence of place image on behavioral intention through attitude.

Based on the above discussion, we hypothesize:

H4: Residents’ place image is positively related to their attitude to tourism.
H5: Residents’ place image is positively related to their pro-tourism behavioral intention.
H5a: Residents’ attitude to tourism positively mediates the relationship between place image and pro-tourism behavioral intention.

Both place image and place attachment have cognitive and affective components and influence behavior (Prayag and Ryan, 2012); Jorgensen and Stedman (2001) even equated place identity and place dependence with cognitive and conative components, respectively. However, research on the link between place attachment and place image is still nascent (Stylidis, 2018).

In Stylidis’s (2018) study, residents with similar place image also had a similar level of place attachment. In Hainan (China), Song et al. (2017) found that golf tourists’ place image of a tourist destination not only directly influenced revisit intention but also indirectly influenced it through place attachment. Using SEM, Tsai (2015) found that destination image positively influenced satisfaction, perceived quality, and emotional place attachment. In a survey of 400 visiting sport event participants, place image affected not only intention to revisit, sport event participation, and recommend but also the level of place attachment (Kaplanidou et al., 2012).

This study uses PLS-SEM to fill the gap in the literature on how residents’ place image indirectly influences their attitude to tourism and pro-tourism behavioral intention through place attachment. Based on the above discussion, we hypothesize:

H6: Residents’ place image is positively related to their place attachment.
H6a: Residents’ place attachment positively mediates the relationship between place image and attitude to tourism.
H6b: Residents’ place attachment positively mediates the relationship between place image and pro-tourism behavioral intention.

**FIGURE 1:**
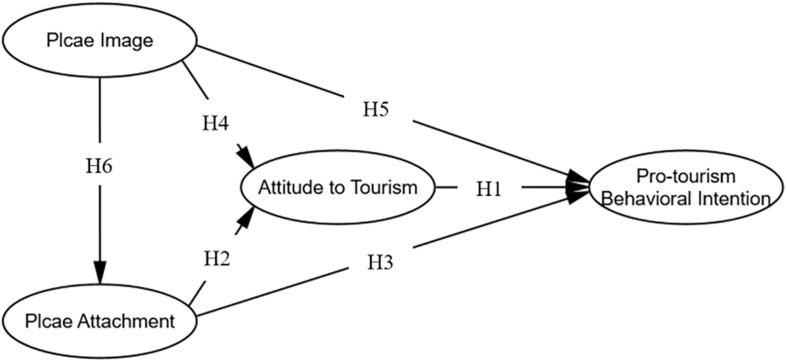
Hypothesized model of residents’ pro-tourism behavioral intention.

Figure 1 demonstrates the hypotheses and relationships between constructs in this study.

## Materials and Methods

### Survey Instrument

All the measurable items were adapted from prior studies. One weakness in the literature is that measures of tourists’ place image have been directly applied for measuring residents’ place image, which “overlooks the multifunctional and daily life world nature of the place for residents” and only covers attractions and amenities, thus ignoring access and ancillary (Stylidis et al., 2016, p. 661). To overcome this deficiency, our study adopts a measurement of place image with four dimensions: community services, physical appearance, social environment, and entertainment services (Stylidis et al., 2014, 2016; Stylidis, 2015). This approach synthesizes both destination and community attributes into the measurement. To assess place attachment, we adapt three items from Tournois and Djeric (2018), whose study area and participants are similar to ours. The original scale demonstrated good reliability (α = 0.84). Attitude to tourism was assessed by four items adapted from Martín et al. (2017). Finally, four items adapted from Ribeiro et al. (2017) were utilized to assess pro-tourism behavioral intention.

Because all the measurement items were originally developed in English, we implemented back-translation (Brislin, 1970): the items were first translated into Chinese by one researcher, and then translated back into English by another researcher. A bilingual speaker checked the translated English to verify that it accurately reproduced the meaning of the original English version.

The whole questionnaire comprised two sections. Section 1 included a total of 24 items for the four constructs in the research model. Participants were asked to respond to each item on a seven-point Likert scale (where 1 = strongly disagree and 7 = strongly agree). Section 2 included six questions on demographics.

### Study Area

Data were collected in Huangshan City, China, located in the south of Anhui Province. Its proximity to Jiangxi, Hubei, Jiangsu, and Zhejiang provinces makes it hold a very important position attracting tourists from home and abroad. Currently, its jurisdiction covers three districts and four counties, with a total area of 9807 km^2^ and a population of about 1.4 million (49.94% male, 50.06% female). About 320,000 residents are aged 18–34 and almost 610,000 are aged 35–59^1^.

As a fifth-tier city, Huangshan City is famous for its tourism resources, with prominent examples including Huangshan Mountains (UNESCO cultural natural heritage and World Geopark), Qiyun Mountain (National Scenic Area, National Geopark, and one of the Four Sacred Taoist Mountains in China), and Gu’niujiang Nature Reserve (National Nature Reserve and National Geopark). With such abundant tourism resources, Huangshan City attracts 10s of 1000s of tourists from across the world annually.

### Sampling and Data Collection

Convenience sampling was conducted from February to March and August to September 2019. Questionnaires were distributed to local residents aged at least 18 years old. Supermarket entrances, streets, and squares were the main places for data collection. An oral filter question was posed before asking each individual to complete the questionnaire, so as to ensure that only residents participated. To seek to ensure response quality, a small gift was offered as a participation reward.

A total of 467 residents were intercepted and asked to complete the questionnaire; 62 refused and 405 obliged. Due to severe missingness or the same rating being given for most questions, 35 questionnaires were discarded. Our final sample for analysis comprised 370 valid questionnaires (see Supplementary Material).

## Results

### Sampling Characteristics

Table 1 profiles the sample. Of the 370 participants, 168 were male (45.4%) and 202 were female (54.6%). More than 70% of participants were married. In terms of age, 83 were 18–25 years old (22.4%), 141 were 26–35 years old (38.1%), and 85 were 36–45 years old (23%), indicating that the majority of participants are young people. Almost half were undergraduates (45.7%), which differs somewhat from the overall population of Huangshan. Regarding jobs, 160 were office workers (43.2%), while the smallest proportion were freelancers (9.5%). The sample was evenly split in terms of income: 45 participants (12.2%) were in the highest-income group (≥¥10,001 per annum), while the income of 103 participants (27.8%) was between ¥4001 and ¥6000. The difference between these two groups was not very sharp.

**TABLE 1:** Descriptive summary of socio-demographic profile.

**Demographic**	**Frequency**	**Percentage**
**Gender (370)**		
Male	168	45.4
Female	202	54.6
**Marital status (370)**		
Single	108	29.2
Married	260	70.3
Others	2	0.5
**Age (370)**		
18–25	83	22.4
26–35	141	38.1
36–45	85	23
46–55	43	11.6
≥ 56	18	4.9
**Education (370)**		
Middle school	71	19.2
Junior college	95	25.7
Undergraduate	169	45.7
Postgraduate	35	9.5
**Job (370)**		
Government agent	72	19.5
Self-employed	48	13
Freelancer	35	9.5
Student	55	14.9
Office worker	160	43.2
**Income (370)**		
≤¥4000	79	21.3
¥4001–6000	103	27.8
¥6001–8000	69	18.6
¥8001–10,000	74	20
≥¥10,001	45	12.2

### Measurement Model and Structural Model

The research model was tested using SPSS 25 and SmartPLS 3.2.8. The high statistical analysis power enables PLS to evaluate the model with a small sample size and non-normal distribution data. It is also suitable for less-developed or exploratory research model (Hair et al., 2012; Ringle et al., 2012). As suggested by Hair et al. (2017), this study followed two steps: assessment of the measurement model (outer) and of the structural model (inner).

In the first stage, the measurement model’s reliability and validity were assessed. As shown in Table 2, factor loadings ranged from 0.798 to 0.901, and thus were all above the threshold of 0.7 suggested by Barclay et al. (1995). Composite reliability and Cronbach’s α values were utilized to assess reliability. They ranged from 0.881 to 0.931 and from 0.797 to 0.919, respectively, thus establishing satisfactory internal consistency (DeVillis, 1991; Hair et al., 2017). We also tested for multicollinearity with the variance inflation factor (VIF). None of the VIFs (inner model and outer model) exceeded 3, suggesting that multicollinearity was not an issue in this study.

**TABLE 2:** Construct reliability and convergent validity.

**Items**	**Factor loading**	**Cronbach’s α**	**CR**	**AVE**
**Attitudes to Tourism**		0.870	0.911	0.719
I believe tourism generates positive benefits for Huangshan City	0.822^∗∗∗^			
I believe tourism is a good activity for Huangshan City	0.874^∗∗∗^			
I would like the tourism sector to continue to play a major role in Huangshan City	0.843^∗∗∗^			
I believe tourism should be actively encouraged in Huangshan City	0.853^∗∗∗^			
**Pro-tourism Behavioral Intention**		0.873	0.913	0.724
I am willing to receive tourists as affable host and being more hospitable	0.885^∗∗∗^			
I am willing to protect the natural and environmental resources on which tourism depends	0.806^∗∗∗^			
I am willing to provide information to tourists and contribute to enhance their experience	0.852^∗∗∗^			
I am willing to do more to promote Huangshan City as tourist destinations	0.858^∗∗∗^			
**Community Services**		0.810	0.888	0.725
Huangshan City has good job opportunities	0.842^∗∗∗^			
Huangshan City has effective government	0.868^∗∗∗^			
Huangshan City has good public transportation	0.844^∗∗∗^			
**Entertainment Services**		0.816	0.891	0.731
Huangshan City has good restaurants	0.833^∗∗∗^			
Huangshan City has good nightlife	0.870^∗∗∗^			
Huangshan City is a good place for shopping	0.862^∗∗∗^			
**Place Attachment**		0.840	0.904	0.758
I will defend Huangshan City when somebody criticizes it	0.811^∗∗∗^			
I will miss Huangshan City when I am not here	0.901^∗∗∗^			
Huangshan City is a part of myself	0.897^∗∗∗^			
**Physical Appearance**		0.846	0.896	0.684
Huangshan City has pleasant weather	0.798^∗∗∗^			
Huangshan City has attractive scenery	0.868^∗∗∗^			
Huangshan City has interesting historic sites	0.818^∗∗∗^			
Huangshan City has nice architecture	0.823^∗∗∗^			
**Social Environment**		0.797	0.881	0.711
Huangshan City is a safe place to live	0.813^∗∗∗^			
Huangshan City is clean	0.877^∗∗∗^			
Huangshan people are friendly	0.839^∗∗∗^			
**Place Image**		0.919	0.931	0.509
Entertainment services	0.810^∗∗∗^			
Social environment	0.841^∗∗∗^			
Physical appearance	0.879^∗∗∗^			
Community services	0.848^∗∗∗^			

*^∗∗∗^*p* < 0.001 level; CR, composite reliability; AVE, average variance extracted.*

Convergent validity was evaluated by average variance extracted (AVE). The AVEs of each construct ranged from 0.509 to 0.758, thus establishing adequate convergent validity (Hair et al., 2014). Discriminant validity was tested with two approaches: Fornell-Larcker criterion analysis and heterotrait-monotrait ratio of correlations (HTMT). As Table 3 shows, the square roots of AVEs on each construct are greater than the correlations between constructs (Fornell and Larcker, 1981). Furthermore, HTMT ratios (in parentheses in Table 3) were all lower than 0.85 (Henseler et al., 2015). The results of these two approaches establish satisfactory discriminant validity.

**TABLE 3:** Correlation matrix of all variables.

**Variables**	**ATT**	**BI**	**CS**	**ES**	**PA**	**PHA**	**SE**
**ATT**	**0.848**						
**BI**	0.642 (0.727)	**0.851**					
**CS**	0.511 (0.606)	0.426 (0.503)	**0.851**				
**ES**	0.478 (0.563)	0.384 (0.454)	0.613 (0.753)	**0.855**			
**PA**	0.541 (0.631)	0.491 (0.571)	0.500 (0.606)	0.520 (0.627)	**0.871**		
**PHA**	0.586 (0.684)	0.439 (0.508)	0.659 (0.792)	0.584 (0.697)	0.522 (0.619)	**0.827**	
**SE**	0.529 (0.637)	0.504 (0.605)	0.616 (0.765)	0.590 (0.727)	0.536 (0.653)	0.655 (0.795)	**0.843**

*ATT, Attitude to Tourism; BI, Pro-tourism Behavioral Intention; CS, Community Services; ES, Entertainment Services; PA, Place Attachment; PHA, Physical Appearance; SE, Social Environment; The bold diagonal elements are the square roots of each AVE; variable correlations are shown off-diagonal. HTMT ratios are in the parentheses.*

**FIGURE 2:**
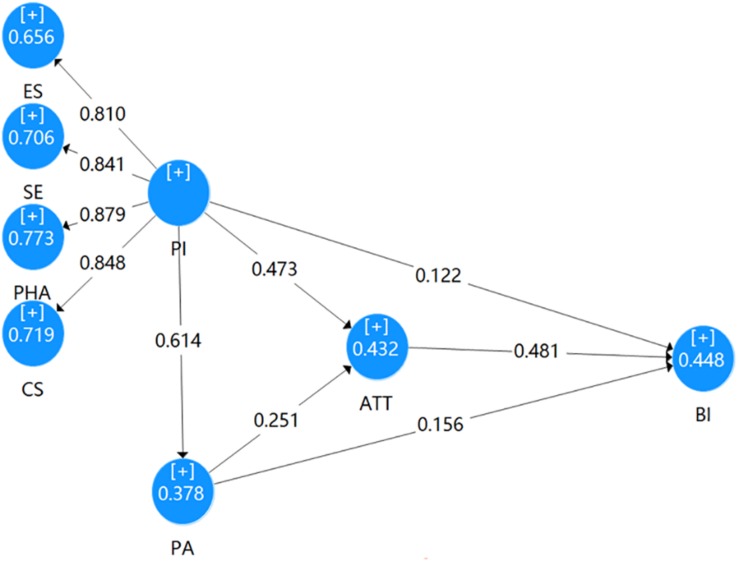
Completely standardized path coefficients among PI, PA, ATT, and BI. ATT, Attitude to Tourism; BI, Pro-tourism Behavioral Intention; PA, Place Attachment; PI, Place Image.

In structural modeling stage, a bootstrapping resampling method (2000 samples) was utilized to assess the statistical significance among variables. Coefficient of determination values (*R*^2^), predictive relevance (*Q*^2^), and path coefficients were selected to assess the structural model. *R*^2^ measures the relationship of a latent variable’s explained variance to its total variance, with a value around 0.333 being considered moderate (Urbach and Ahlemann, 2010). Thus, *R*^2^ values (Attitude: 0.432; Behavioral intention: 0.448; Place attachment: 0.378) of this study are satisfactory (see Figure 2). *Q*^2^ measures the extent to which each prediction is successful. The *Q*^2^ value of each endogenous variable was positive (Attitude: 0.292; Behavioral intention: 0.298; Place attachment: 0.271), thus confirming the model’s predictive relevance in respect of a particular construct (Urbach and Ahlemann, 2010).

Table 4 reports the estimated path coefficients between variables in the research model. Residents’ attitude to tourism was found to positively and significantly affect pro-tourism behavioral intention (β = 0.481, *p* < 0.001), thus supporting H1. Place attachment was found to positively and significantly influence residents’ attitude to tourism (β = 0.251, *p* < 0.001), as predicted by H2. Place attachment significantly impacted on pro-tourism behavioral intention (β = 0.156, *p* = 0.010), thus supporting H3. Also, place image was found to positively and significantly relate to residents’ attitude to tourism (β = 0.472, *p* < 0.001) and place attachment (β = 0.614, *p* < 0.001), thus respectively supporting H4 and H6. However, place image was found to have no significant impact on pro-tourism behavioral intention (β = 0.122, *p* = 0.062), so H5 was rejected.

**TABLE 4:** Results of the structural model.

**Hypotheses**	**Path**	**Original sample**	**Standard error**	***t*-Value**	***p*-Value**	**Support**
H1	ATT→BI	0.481	0.060	8.037	0.000	Yes
H2	PA→ATT	0.251	0.062	4.043	0.000	Yes
H3	PA→BI	0.156	0.061	2.571	0.010	Yes
H3a	PA→ATT→BI	0.121	0.032	3.825	0.000	Yes
H4	PI→ATT	0.473	0.063	7.480	0.000	Yes
H5	PI→BI	0.122	0.065	1.870	0.062	No
H5a	PI→ATT→BI	0.227	0.045	5.108	0.000	Yes
H6	PI→PA	0.614	0.034	17.973	0.000	Yes
H6a	PI→PA→ATT	0.154	0.038	4.076	0.000	Yes
H6b	PI→PA→BI	0.096	0.038	2.550	0.011	Yes

*ATT, Attitude to Tourism; BI, Pro-tourism Behavioral Intention; PA, Place Attachment; PI, Place Image.*

To test the mediation effect of residents’ place attachment and attitude to tourism, we utilized a bootstrapping resampling approach (2000 samples). As demonstrated in Table 4, residents’ place attachment was found to indirectly and positively affect pro-tourism behavioral intention through their attitude to tourism (β = 0.121, *p* < 0.001), thus supporting H3a. Residents’ place image was found to indirectly and positively impact pro-tourism behavioral intention through their attitude to tourism (β = 0.227, *p* < 0.001), thus supporting H5a. It was also confirmed that residents’ place image indirectly affected attitude to tourism and pro-tourism behavioral intention, respectively, through place attachment (β = 0.154, *p* < 0.001; β = 0.096, *p* = 0.011), thus supporting H6a and H6b. Besides, the direct effect of residents’ place image on their pro-tourism behavioral intention was not statistically significant, suggesting that residents’ attitude to tourism and place attachment fully mediated between place image and pro-tourism behavioral intention. Also, the statistically significant direct effect of place image on residents’ attitude to tourism indicates that place attachment partially mediated this relationship. Lastly, the statistically significant direct effect of residents’ place attachment on their pro-tourism behavioral intention indicates that attitude to tourism partially mediated this relationship.

## Discussion

This study aimed to deepen understanding of residents’ pro-tourism behavioral intention by analyzing how place image and place attachment influence their attitude to tourism and, ultimately, their behavioral intention. Nine of the 10 hypothesized relationships were supported, which provides valuable insights for researchers and practitioners.

Our finding of a direct positive relationship between attitude to tourism and pro-tourism behavioral intention (supporting H1) accords with TPB, TRA, and TAM, as well as considerable previous studies. Among other scholars, Lepp (2007) previously found that residents with a positive attitude to tourism have higher pro-tourism behavioral intention (Martín et al., 2017; Moghavvemi et al., 2017). There is, thus, strong evidence that attitude is an important precondition for performing a given behavior.

The positive relationship between place attachment and attitude to tourism (confirming H2) is in line with the findings of Lee (2013) and Eusébio et al. (2018). Eusébio et al. (2018) found that the higher the destination attachment, the more positive were residents’ attitude to tourism. Lee (2013) drew the similar conclusion that residents’ attachment to their community significantly influenced their attitude to sustainable tourism development.

Our finding of a positive relationship between residents’ place attachment and pro-tourism behavioral intention (supporting H3) reinforces previous studies’ results (Gursoy and Rutherford, 2004; Nicholas et al., 2009). Lee (2013) confirmed that higher community attachment among residents resulted in stronger support for tourism development. Choi and Murray (2010) also found that highly attached residents appear to positively evaluate tourism development. These consistent findings suggest that stronger place attachment among residents brings greater support for tourism. High place attachment means residents are more dependent on the place; accordingly, since tourism development can generate benefits for local residents, those with strong place attachment are more inclined to support it.

Place image positively influenced residents’ attitudes to tourism and place attachment (respectively supporting H4 and H6), which is also consistent with previous studies. Stylidis (2018) found that *Appreciators* (residents with the most favorable place image) reported high place attachment, while the *Critical* (residents with the least favorable place image) also reported low place attachment. Similarly, Stylidis (2017) reported a positive relationship between residents’ place image and place attachment, and that residents’ place image positively affected their attitude to tourism (Ramkissoon and Nunkoo, 2011). Combined, these findings confirm that the more positive the residents’ place image, the higher their place attachment and the more positive their attitude to tourism.

Surprisingly, the positive effect of place image on pro-tourism behavioral intention predicted by H5 was not found to be statistically significant, contradicting the results of previous studies (Ramkissoon and Nunkoo, 2011; Stylidis et al., 2014; Stylidis, 2015). In Port Louis, Ramkissoon and Nunkoo (2011) found that every place image dimension except government service influenced residents’ level of support for the tourism industry. However, whereas they used four types of attributes to measure place image (social, transport, government services, and shopping), our four-dimension measurement was more comprehensive. Based on data collected in Kavala, Stylidis (2015) confirmed that two dimensions of place image (physical appearance and social environment) positively affected residents’ support for tourism. With the same data, Stylidis et al. (2014) also found that residents’ place image as a holistic construct affected their support for tourism. The difference between our result and theirs may be attributable to differences between the focal cities and the attributes of their respective residents. Greece is a developed country, while China is developing; the local economy of Kavala is mainly based on extraction and export of natural resources, whereas the main resource for (fifth-tier) Huangshan City is tourism. Furthermore, our sample included permanent residents and some immigrants temporarily residing for work, whereas Stylidis et al. (2014) and Stylidis (2015) only studied permanent residents. This may explain different perceptions of place image and levels of support toward tourism among participants.

Regarding H3a, H5a, H6a, and H6b, the predicted mediating effects of residents’ attitude and place attachment were all supported. The mediating influence was an exploratory test and found significant in this study, which demonstrates the complicated relationship among place image, place attachment, attitude to tourism, and pro-tourism behavioral intention. It is noteworthy that attitude to tourism and place attachment fully mediate between place image and pro-tourism behavioral intention. In other words, place image would not have impacted pro-tourism behavioral intention without attitude to tourism or place attachment. In addition, place attachment’s partial mediation between place image and attitude to tourism suggests that place attachment reinforced the impact of place image on attitude to tourism. Lastly, attitude to tourism’s partial mediation between place attachment and pro-tourism behavioral intention indicates that a more favorable attitude strengthens the impact of place attachment on support for tourism.

## Theoretical and Practical Implications

By examining the relationship among residents’ place image, place attachment, attitude to tourism, and pro-tourism behavioral intention, this study contributes to the scholarly fields of tourism and environmental psychology. In congruence with the hypothesized relationships, results revealed that residents’ place attachment positively and directly influenced their attitude to tourism and pro-tourism behavioral intention, as well as indirectly influencing pro-tourism behavioral intention via attitude to tourism. This extends limited existing evidence on place attachment as a direct and indirect predictor of residents’ pro-tourism behavioral intention (Eusébio et al., 2018). Moreover, this study extends the literature on residents’ place image (Stylidis et al., 2016, 2018) and demonstrates its impact on their place attachment. Contrary to prior studies, the impact of place image on residents’ pro-tourism behavioral intention is not significant, indicating the need to further investigate this relationship. Importantly, this is the first study to examine the indirect impact of place image on residents’ attitude to tourism through place attachment, and future studies should further pursue this line of enquiry.

Place attachment and attitude are key constructs respectively developed in environmental psychology and social psychology (Altman and Low, 1992; Gu and Ryan, 2008; Fornara et al., 2019). Moreover, environmental psychology research has long acknowledged the significant impact of place image on residents’ attitude to tourism and behaviors (Stylidis, 2015). As interdisciplinary variables, we found strong empirical support for their ability to predict residents’ attitude to tourism and pro-tourism behavioral intention, both directly and indirectly. Some scholars suggest that environmental psychology should only concentrate on goal-directed behavior (Kaiser and Wilson, 2004). Accordingly, this study’s attempt to examine the correlations among the four variables contributes to extending and conceptually linking the literature on environmental psychology and tourism, thus innovating through cross-disciplinary insights.

This study’s implications can also guide the improvement of local tourism development. Though residents’ place image was not directly related to their pro-tourism behavioral intention, it could indirectly affect it through their attitudes to tourism. Therefore, to gain residents’ support for tourism development, the local tourism sector should concentrate on improving a destination’s image and residents’ attachment to their environment. Potentially valuable initiatives include focusing on the “green” dimension to enhance place attachment (Scannell and Gifford, 2010), creating more job opportunities, and improving government efficiency, transportation, and community services to enhance the positive image for local residents. As residents’ place image was also related to their place attachment, place image is key to gaining support from local residents.

In sum, more actions should be taken to improve residents’ place image and place attachment to gain their support for tourism development and ultimately maintain tourism sustainability.

## Limitations and Future Research

The first limitation is generalizability. The study used data from a fifth-tier city, so it is questionable whether similar results would be found in other cities, such as Beijing or Shanghai. In addition, the characteristics of the sample differ somewhat from those of the wider population of study area, so with a more representative sample, the strength of the relationships between the four variables may vary. The second limitation is that every participant was recruited in the most crowded and popular places, such as supermarket entrances, streets, and squares. Therefore, the opportunity to participate may not have been the same for all local residents. The third limitation is that we only considered the influence of two exogenous variables on attitude and behavioral intention. More variables should be considered in the model to form a better understanding of residents’ pro-tourism behavioral intention, such as seasonality (Vargas-Sánchez et al., 2013), empowerment (Boley et al., 2014), and emotional solidarity (Woosnam, 2012). Residents’ attitude to tourism and consequent behavioral intention are affected by differences in empowerment, emotional solidarity, and between high and low season. Moreover, some potentially moderating factors should also be considered, such as tourism-related job and socio-demographic information. For instance, residents who work in the hospitality and tourism industry will perceive tourism more positively and be more likely to support it than residents outside this industry. Finally, attitude to tourism is too general: future studies should break it down into several categories, such as attitude to tourism, to behavior, and to tourists.

## Data Availability Statement

All datasets generated for this study are included in the manuscript/Supplementary Files.

## Ethics Statement

Ethical review and approval was not required for the study on human participants in accordance with the local legislation and institutional requirements. Written informed consent for participation was not required for this study in accordance with the national legislation and the institutional requirements.

## Author Contributions

KS and CG conceived the study. KS, CG, and XS wrote the manuscript. All authors designed the study, collected and analyzed the data, read and approved the manuscript, and agreed to be accountable for all aspects of the work.

## Conflict of Interest

The authors declare that the research was conducted in the absence of any commercial or financial relationships that could be construed as a potential conflict of interest.

## Footnotes

1 http://zw.huangshan.gov.cn/OpennessContent/show/1289624.html?tdsourcetag=s_pcqq_aiomsg

## Supplementary Material

TABLE S1 Raw data of 370 participants.
